# From Epiphenomenon to Biologically Important Phenomena

**DOI:** 10.1017/pen.2017.1

**Published:** 2018-05-25

**Authors:** Philip J Corr, Dean Mobbs

**Affiliations:** 1 Founding Editor-in-Chief; 2 Assistant Editor

The successful attempt to explain brain–behavioral relationships heralded a revolution in cognitive neuroscience in the mid-to-late 20th century. Some notable examples stand out. David Hubel and Torsten Wiesel showed that firing patterns of cells in the visual cortex reflect simple elements of the visual input, including orientation, as well as sensory deprivation early in life prevents neurogenesis in the visual cortex with irreparable consequences for the visual system. Brenda Milner discovered that hippocampus resection results in anterograde amnesia. James Olds and Peter Milner showed that rats will self-stimulate reward circuits that control the reinforcement of behavior. However, as in other related areas (e.g., experimental/cognitive psychology), cognitive neuroscience often treated discussion of individual differences as noise or epiphenomenon—although there are exceptions, such as individual differences in the reinforcing properties of self-administered drugs (Piazza et al., 1991) and the study of emotionality (Blizard, 1981; Broadhurst, 1975).

By the turn of the 21st century, neuroscientists started to discover relationships between neural circuits and behavior that exhibited surprising variability. Eve Marder (Weimann & Marder, 1994) showed how different networks can produce the same behavior (e.g., circuits of crustaceans can generate similar neuronal outputs using multiple configurations). Jeff Lichtman showed how networks that are substantially different within species can produce the same behavior (Morgan & Lichtman, 2013). Differences between transgenic mice (e.g., C57BL/6 vs. BALB/c strains) were shown to have implications for human clinical conditions, the underlying phenotypes of which are known to show considerable individual variation (e.g., clinical anxiety; Sartori, Landgraf, & Singewad, 2011; see also Hefner et al., 2008). A critical discovery was that subtle effects of environmental context can translate into large individual differences in behavior (Crabbe, Wahlsten, & Dudek, 1999). Turning to human studies, neuroscientists showed, among other things, how brain damage to the same area can result in different behaviors between people (e.g., Mobbs, Lau, Jones, Frith, 2007); and how experience changes the structure of the brain (Maguire et al., 2000). The contemporary view is that neural circuits are complex, plastic, dynamic, often closely coupled with the environment, and remarkably diverse with species.

Around the same time, the field of personality psychology was witnessing its own revolution. Theorists began to move away from consideration of whether traits exist or not (everyone began to agree that they do), to an exploration of their biological nature—although it was still uncommon to see personality discussed in biological psychology textbooks (for an exception, see Corr, 2006). Taking his lead from Ivan Pavlov—whose attempt to unite personality, psychiatry, and neurophysiology still influences psychology and neuroscience—Hans Eysenck ploughed his own, somewhat lonely, furrow from the early 1940s (1944, 1947) to launch his 1967 fully fledged biological theory of personality (for a summary, see Corr, 2016). This was soon followed by the seminal work of one of Eysenck’s students, Jeffrey Gray, who coopted sophisticated learning theory, neuropharmacology, and brain techniques to develop a true neuropsychology of emotion, motivation, and personality (Gray, 1970, 1972, 1987). This work culminated in the characterization of the Behavioral Inhibition System model of anxiety (Gray, 1982; Gray & McNaughton, 2000). Gray’s general approach—which, itself, traces its origins to Pavlov (Gray, 1964)—has proliferated in personality psychology and is now a major scientific force (see Corr, DeYoung, & McNaughton, 2013). During this period, there was also an explosion of research on the “Big-Five” model of personality and it is now increasingly studied from a neuroscience perspective (e.g., Allen & DeYoung, 2017)—this fact is attested by the first paper published in this journal by Nicola Toschi and colleagues which is on the functional connectome of the five-factor model of personality.

In addition to these significant advances, we now know that personality differences have important implications for a wide range of behaviors, including clinical disorders, occupational choice/performance, judgment and decision making, health behavior—including personality contributions to mortality (e.g., Bogg & Roberts, 2004; Calvin et al., 2017)—and much more besides. These advances have influenced related fields, some of which are of widespread relevance to society (e.g., behavioral and neuroeconomics; for an overview, see the edited book by Reuter & Montag, 2016).

Personality neuroscience as an independent field of enquiry gained in prominence when it became evident that systematic individual differences could be mapped jointly to behavior and neural activity. One of the first studies in the field came from Canli, Sivers, Whitfield, Gotlib, and Gabrieli (2002), who published a paper in *Science* reporting that extraversion is associated with an elevated BOLD signal in the amygdala while viewing happy, but not fearful, faces. This study was soon followed by research coupling behavioral genetics with brain imaging: Several studies were published in high-profile journals reporting that specific genotypes (e.g., 5HTTLPR short vs. long allele polymorphisms) might be associated with differential amygdala reactivity (e.g., Pezawas et al., 2005; note the seminal work on 5-HTTLPR published earlier by Lesch et al., 1996). Around the same time, Passamonti et al. (2006) were the first to show how the MAOA gene (implicated in the catabolism of serotonin) is related to brain function associated with impulsive personality traits. Although these and other studies were critiqued as underpowered and statistically unreliable (Kriegeskorte, Simmons, Bellgowan, and Baker, 2009; Vul, Harris, Winkielman, & Pashler, 2009), they laid the path for more powerful ones that, for example, support: (a) the notion that gene variation and expression reliably predict alteration in functional brain networks (Richiardi, Altmann, Milazzo, Chang, & Chakravarty, 2015); (b) stable connectivity patterns predict cognitive ability (Finn et al., 2015); and (c) structural brain differences predict psychiatric disorder (e.g., Hazlett, Gu, & The IBIS Network, 2017). These and other recent studies took advantage of the critical self-examination in the field, of improved statistical approaches, and of the availability of shared data sets with considerably larger sample sizes—something that is now especially seen in genome-wide association studies of personality (de Moor et al., 2012; van den Berg et al., 2016).

No doubt, much of the excitement in personality neuroscience arises from recent discoveries in molecular genetics, where polygenic contributions to personality traits (e.g., Luciano et al., 2018) and cognitive abilities (e.g., Hill et al., 2018) have been identified. This has led to definite suggestions as to their causal bases, and this has included cross-cultural work related to clinically relevant phenotypes (e.g., 5-HTTLPR and neuroticism; Montag & Reuter, 2014). With large samples sizes and robust methods, reproducibility and replication are becoming less of a problem.

The inception of *Personality Neuroscience* coincides with these significant empirical and methodological developments; and so too with the recognition of some broader conceptual issues. For example, as highlighted by Luca Passamonti (2018, personal communication), in common with cognitive neuroscience more generally, the issue of “reverse inference” needs to be recognized: Reasoning “back” from brain functional activations (or structural changes) to an underlying cognitive or affective state that has not been directly measured (although this may be less of a problem than it might seem; see Hutzler, 2014; Poldrack, 2006). To address this issue, more formal ways to infer mental states from neuroimaging results and novel analytical tools (including multivoxel pattern analysis and multivariate decoding) have been developed within the field of machine learning (Poldrack, 2011). However, as these approaches are correlational, older approaches, such as lesion studies (especially those which temporally associate a brain lesion with a particular behavioral change), and newer methodologies, such as transcranial magnetic stimulation, remain essential to attribute a causal role to a network or a brain region in relation to psychological states and processes.

## Editorial Board Opinions

Driven by the above advances, and many more besides, *Personality Neuroscience* was established to consolidate and strengthen the field. The journal is supported by an Editorial Board of leading scientists who represent a broad constituency of theory, method, and applications. It is sad though that one of the leading lights of the field and a Founding Editorial Board member, Jaak Panksepp, did not live long enough to see its launch. It is appropriate that the journal contains his obituary as well as a paper summarizing his seminal contributions over many years.

Given the involvement of the Editorial Board in shaping the scope and policies of the journal, it is of some interest to consider their views on the nature of personality neuroscience—they go a long way to defining the field. At a general level, one Editorial Board member states succinctly: “As any neuroscientist knows, no two brains are identical.” *Personality Neuroscience* aims to answer how and why this is the case, and the implications for behavior and experience. A common theme expressed by several Board members is that, despite the fact that neuroscience has dominated psychological research and funding for the last 25 years, it has largely ignored issues of systematic differences between individuals. However, the scientific climate is changing, as noted by another Editorial Board member: “… after decades of semi-neglect, personality traits are edging their way into biologically-informed psychiatrists’ minds as tractable indicators of liability to psychiatric disorders ….Yes, if only they had listened to Hans Eysenck.” Of course, the use of low-powered studies, especially with small sample sizes made this semineglect almost inevitable; but, more recently, neuroscientists have come to appreciate that they cannot continue to ignore the systematic ways in which their study participants vary—or, if they do, they run the risk of discarding scientifically valuable information. This is especially true in the era of large-scales studies (e.g., genome-wide association studies; see above) that are providing robust insights into the neurobiological mechanisms of individual differences. We now know that personality traits can be studied in novel ways, including functional connectomics, large-scale neurogenomics, mechanistic studies of the molecular basis of gene–environment interactions, and social regulation of gene expression.

It is evident from the comments of the Editorial Board that there is much interest in the potential of the field to make meaningful contributions to psychiatry and psychological well-being more generally. This is an important topic, especially when seen in the light of the early work that had the aim of uniting personality and psychopathology within a single theoretical framework. An early example was Hans Eysenck’s (1944) factor analytical study of 700 “war neurotics”. in which he statistically isolated Introversion-Extraversion and Stability-Neuroticism, subsequently relating them to major clusters of internalizing and externalizing disorders within an overarching theory (Eysenck, 1947; see also Eysenck & Eysenck, 1985). As presaged by this early research, we now know that personality factors (e.g., Neuroticism and Conscientiousness) are implicated in modifying the risk of developing a broad range of psychiatric conditions, including anxiety and depressive disorders (Genetics of Personality Consortium et al., 2015; Kern & Friedman 2011)—two of the heaviest health burdens on society today.

However, a challenge facing the field is the continued reliance on self-report of individual differences, and in clinical psychology often of diagnosis. This state of affairs is not helped by the paucity of strong neuroscientific theories that have the capacity to connect with the biology underlying normal and abnormal trait variations: Biologically informed endophenotypes are clearly needed. But, novel approaches are emerging, such as “behavioral signatures” of personality, that hold considerable potential. This is seen with a younger generation of researchers interested in digital phenotyping (e.g., social media footprints; Azucar, Marengo, & Settanni, 2018) who apply methods from psychoinformatics (Yarkoni, 2012; Markowetz, Błaszkiewicz, Montag, Switala, & Schlaepfer, 2014). This type of information can be combined with neuroscientific data, potentially merging into a discipline called “psychoneuroinformatics” (Montag & Diefenbach, 2018).

As noted by several Editorial Board members, one major outcome of discovering the neuroscience roots of personality traits is the potential for designing personalized therapeutic approaches and individualized health care (Finn & Constable, 2016)—this is the promise of “precision medicine”, “gene editing technologies”, and identification of “personality signatures” of neural measures. This is especially relevant in clinical psychopharmacology, as patients respond very differently to the same drugs. Although there are several reasons (e.g., metabolism) for these response differences, an often neglected one is personality differences which makes sense when they are seen as reflecting the underlying variability in brain structure and function. In this regard, it is of no small interest to note that psychoactive drugs have long been used to test personality processes (e.g., in the work of Jeffrey Gray, 1977, and Eysenck’s, 1963, “experiments with drugs”)—indeed, Gray’s (1982) behavioral dissection of defensive systems was based on rats’ differential responses to classes of drugs used in the human clinic (e.g., anxiolytics; see McNaughton & Corr, 2018). Putting all of the above together, as one Editorial Board member aptly characterizes the field: “We seem to have a renais*science* on our hands.”

However, it is also appropriate to sound a note of caution: We need to resist getting carried away with the apparent potential of the field. It is necessary that we recognize major problems and ensure that research and its publication are appropriately tempered. We need to remain self-reflective and critical, and avoid being too Panglossian in outlook. As one Editorial Board member observes: “One important issue *Personality Neuroscience* might be able to tackle is finally to make it clear that personality might not necessarily come together in some neat mechanistic way in the brain and that trying to reduce it to collections of ‘traits’ based on factor analysis is not helpful.” Opinions differ on such matters and this is a health state of affairs.

## Editorial policy

Given the great leaps in methodology, especially in the age of the availability of large databases and theoretical advances, it is timely to create a journal dedicated to the neuroscience of individual variation in brain and behavior. *Personality Neuroscience* aims to provide a high-impact publication outlet for research devoted to understanding the *causal* basis of personality—broadly interpreted, including biology, and biology–environment (including social) interactions. It will cover a wide spectrum of individual differences; one that is not limited to a narrow definition of personality, but includes cognitive abilities, emotionality, and related psychological processes. Submissions to *Personality Neuroscience* entailing the application to areas as clinical disorders, health, behavioral and neuroeconomics, and even workplace behavior, are encouraged: The journal accords basic theory and translational applications equal status.

Empirical papers focussed on the interface between personality and neuroscience are especially welcomed—these can take various forms (e.g., experimental, longitudinal, genetic, genomic, gene expression, and epigenetic). Cross-sectional and largely correlational studies will be considered only if they are highly robust, well-powered, and innovative—and sufficiently related to the issue of *causal* processes (e.g., a psychometric tool designed to explore biological factors in personality). Nonhuman animal studies will be published, as will clinical research—the journal recognizes the importance of “bench to bedside” knowledge.

*Personality Neuroscience* aims to publish only the highest quality work in personality neuroscience, but they need not be *apparently* perfect—we know that science does not work that way. The journal insists upon “honest” research: Papers that contain a “warts-and-all” approach, with limitations and caveats not only acknowledged but highlighted. The overriding criterion for publication is that work has the potential to advance the field and this can take different forms, including identification and clarification of thorny conceptual, theoretical, or methodological problems. Theory papers and systematic reviews are welcomed, especially ones that consolidate the extant research literature, and occasional letters on topical issues will also be considered.

Through its focus on the *equal* importance of personality and neuroscience, the overriding aim of *Personality Neuroscience* is to enable the publication of personality neuroscientists’ work: No longer is there the need to trade-off one side of the scientific coin for the other.

## References

[ref1] Allen T. A. DeYoung C. G. (2017). Personality neuroscience and the five factor model In T. A. Widiger (Ed.), Oxford handbook of the five factor model (pp. 319–352). New York: Oxford University Press.

[ref2] Azucar D., Marengo D. Settanni M. (2018). Predicting the Big 5 personality traits from digital footprints on social media: A meta-analysis. Personality and Individual Differences, 124, 150–159. 10.1016/j.paid.2017.12.018.

[ref3] Blizard D. A. (1981). The Maudsley reactive and nonreactive strains: A North American perspective. Behavior Genetics, 11, 469–489. 10.1007/BF01070004. 7325951

[ref4] Bogg T. Roberts B. W. (2004). Conscientiousness and health-related behaviors: A meta-analysis of the leading behavioral contributors to mortality. Psychological Bulletin, 130, 887–919. 10.1037/0033-2909.130.6.887. 15535742

[ref5] Broadhurst P. L. (1975). The Maudsley reactive and nonreactive strains of rats: A survey. Behavior Genetics, 5, 299–319. 10.1007/BF01073201. 1191155

[ref6] Calvin C. M., Batty G. D., Der G, Brett C. E., Taylor A, Pattie A., & Deary I. J. (2017). Childhood intelligence in relation to major causes of death in 68 year follow-up: Prospective population study. British Medical Journal, 357, 2708 10.1136/bmj.j2708. PMC5485432 28659274

[ref7] Canli T., Sivers H., Whitfield S. L., Gotlib I. H. Gabrieli J. D. (2002). Amygdala response to happy faces as a function of extraversion. Science, 296, 2191 10.1126/science.1068749. 12077407

[ref8] Corr P. J. (2006). Understanding biological psychology. Oxford: Blackwell.

[ref9] Corr P. J. (2016). Hans Eysenck: A contradictory psychology (Mind Shapers series). London: Palgrave.

[ref10] Corr P. J., DeYoung C. G. McNaughton N. (2013). Motivation and personality: A neuropsychological perspective. Social and Personality Psychology Compass, 7, 158–175. 10.1111/spc3.12016.

[ref11] Crabbe J. C., Wahlsten D. Dudek B. C. (1999). Genetics of mouse behavior: Interactions with laboratory environment. Science, 284, 1670–1672. 10.1126/science.284.5420.1670. 10356397

[ref12] de Moor M. H, Costa P. T., Terracciano A., Krueger R. F., de Geus E. J. C., Toshiko T., & Boomsma D. I. (2012). Meta-analysis of genome-wide association studies for personality. Molecular Psychiatry, 17, 337–349. 10.1038/mp.2010.128. 21173776 PMC3785122

[ref19] de Moor M. H. M., van den Berg S. M., Verweij K. J. H., **&** **Genetics of Personality Consortium**. (2015). Meta-analysis of genome-wide association studies for neuroticism, and the polygenic association with major depressive disorder. JAMA Psychiatry, 72, 72642–72650. 10.1001/jamapsychiatry.2015.0554. PMC4667957 25993607

[ref13] Eysenck H. J. (1944). Types of personality: A factorial study of seven hundred neurotics. Journal of Mental Science, 90, 851–861. 10.1192/bjp.90.381.851.

[ref14] Eysenck H. J. (1947). Dimensions of personality. London: Kegan Paul.

[ref15] Eysenck H. J. (Ed.) (1963). Experiments with drugs. Oxford: Macmillan.

[ref16] Eysenck H. J. (1967). The biological basis of personality. Springfield, IL: Charles C. Thomas.

[ref60] Eysenck H. J. Eysenck M. W. (1985). Personality and individual differences: A natural science approach. New York: Plenum.

[ref17] Finn E. S. Constable R. T. (2016). Individual variation in functional brain connectivity: Implications for personalized approaches to psychiatric disease. Dialogues in Clinical Neuroscience, 18, 277–287. 27757062 10.31887/DCNS.2016.18.3/efinn PMC5067145

[ref18] Finn E. S., Shen X., Scheinost D., Rosenberg M. D., Huang J., Chun M. M., & Constable R. T. (2015). Functional connectome fingerprinting: Identifying individuals using patterns of brain connectivity. Nature Neuroscience, 18, 1664–1671. 10.1038/nn.4135. 26457551 PMC5008686

[ref20] Gray J. A. (1964). Pavlov’s typology. Oxford: Pergamon Press.

[ref21] Gray J. A. (1970). The psychophysiological basis of introversion-extraversion. Behaviour Research and Therapy, 8, 249–266. 10.1016/0005-7967(70)90069-0. 5470377

[ref22] Gray J. A. (1972). Learning theory, the conceptual nervous system and personality In V. D. Nebylitsyn, & J. A. Gray (Eds.), The biological bases of individual behaviour (pp. 372–399). London, New York: Academic Press.

[ref23] Gray J. A. (1977). Drug effects on fear and frustration: Possible limbic site of action of minor tranquilizers In L. L. Iversen, S. D. Iversen, & S. H. Snyder (Eds.), Handbook of psychopharmacology. Vol 8: Drugs, neurotransmitters and behaviour (pp. 433–529). New York: Plenum Press.

[ref24] Gray J. A. (1982). The neuropsychology of anxiety: An enquiry in to the functions of the septo-hippocampal system. Oxford: Oxford University Press.

[ref25] Gray J. A. (1987). The psychology of fear and stress. London: Cambridge University Press.

[ref26] Gray J. A. McNaughton N. (2000). The neuropsychology of anxiety: An enquiry into the functions of the septo-hippocampal system (2nd ed.), Oxford: Oxford University Press.

[ref27] Hazlett H. C. Gu H., The IBIS Network (2017). Early brain development in infants at high risk for autism spectrum disorder. Nature, 542, 348–351. 10.1038/nature21369. 28202961 PMC5336143

[ref28] Hefner K., Whittle N., Juhasz J., Norcross M., Karlsson R-M, Saksida L. M, & Holmes A. (2008). Impaired fear extinction learning and cortico-amygdala circuit abnormalities in a common genetic mouse strain. The Journal of Neuroscience, 28, 8074–8085. 10.1523/JNEUROSCI.4904-07.2008. 18685032 PMC2547848

[ref29] Hill W. D., Marioni R., Maghzian O., Ritchie S., Hagenaars S. P., McIntosh A. M., & Deary I. J. (2018). A combined analysis of genetically correlated traits identifies 187 loci and a role for neurogenesis and myelination in intelligence. *Molecular Psychiatry*, 10.1038/s41380-017-0001-5. PMC6344370 29326435

[ref30] Hutzler F. (2014). Reverse inference is not a fallacy per se: Cognitive processes can be inferred from functional imaging data. Neuroimage, 84, 1061–1069. 10.1016/j.neuroimage.2012.12.075. 23313571

[ref31] Kern M. L. Friedman H. S. (2011). Personality and pathways of influence on physical health. Social and Personality Psychology Compass, 5, 76–87. 10.1111/j.17519004.2010.00331.x.

[ref32] Kriegeskorte N., Simmons W. K., Bellgowan P. S. F. Baker C. I. (2009). Circular analysis in systems neuroscience: The dangers of double dipping. Nature Neuroscience, 12, 535–540. 10.1038/nn.2303. 19396166 PMC2841687

[ref33] Lesch K. P., Bengel D., Heils A., Sabol S. Z., Greenberg B. D., Petri S., & Murphy D. L. (1996). Association of anxiety-related traits with a polymorphism in the serotonin transporter gene regulatory region. Science, 274, 1527–1531. 10.1126/science.274.5292.1527. 8929413

[ref34] Luciano M., Hagenaars S. P., Davies G., Hill W. D., Clarke T-K., Shirlai M., & Deary I. J. (2018). 116 independent genetic variants influence the neuroticism personality trait in over 329,000 UK Biobank individuals. Nature Genetics, 50, 6–11. 10.1038/s41588-017-0013-8. 29255261 PMC5985926

[ref35] Maguire E. A., Gadian D. G., Johnsrude I. S., Good C. D., Ashburner J., Frackowiak R. S., & Frith C. D. (2000). Navigation-related structural change in the hippocampi of taxi drivers. Proceedings of the National Academy of Sciences, 97, 4398–4403. 10.1073/pnas.070039597. PMC18253 10716738

[ref36] Markowetz A., Błaszkiewicz K., Montag C., Switala C. Schlaepfer T. E. (2014). Psycho-informatics: Big data shaping modern psychometrics. Medical Hypotheses, 82, 405–411. 10.1016/j.mehy.2013.11.030.. 24529915

[ref37] McNaughton N. Corr P. J. (2018). Survival circuits and risk assessment. In D. Mobbs & J. LeDoux (Eds.), Survival circuits. Current Opinion in Behavioral Sciences (Special Issue), 24, 14–20. 10.1016/j.cobeha.2018.01.018.

[ref38] Mobbs D., Lau H. C., Jones O. D. Frith C. D. (2007). Law, responsibility and the brain. PLoS Biology, 5, E103 10.1371/journal.pbio.0050103. 17439297 PMC1852146

[ref39] Montag C. Diefenbach S. (2018). Towards homo digitalis: Important research issues for psychology and the neurosciences at the dawn of the internet of things and the digital society. Sustainability, 10, 415 10.3390/su10020415.

[ref40] Montag C. Reuter M. (2014). Disentangling the molecular genetic basis of personality: From monoamines to neuropeptides. Neuroscience & Biobehavioral Reviews, 43, 228–239. 10.1016/j.neubiorev.2014.04.006. 24769290

[ref41] Morgan J. L. Lichtman J. W. (2013). Why not connectomics? Nature Methods, 10, 494–500. 10.1038/nmeth.2480. 23722208 PMC4184185

[ref42] Passamonti L., Fera F., Magariello A., Cerasa A., Gioia M. C., Muglia M., & Quattrone A. (2006). Monoamine oxidase-A genetic variations influence brain activity associated with inhibitory control: New insight into the neural correlates of impulsivity. Biological Psychiatry, 59, 334–340. 10.1016/j.biopsych.2005.07.027. 16202396

[ref43] Piazza P. V., Maccari S., Deminiere M., Moal H. L., Mormede P. Simon H. (1991). Corticosterone levels determine individual vulnerability to amphetamine self-administration. Proceedings of the National Academy of Sciences, 88, 2088–2092. 10.1073/pnas.88.6.2088. PMC51174 2006148

[ref44] Pezawas L, Meyer-Lindenberg A., Drabant E. M., Verchinski B. A., Munoz K. E., Kolachana B. S., & Weinberger D. R. (2005). 5-HTTLPR polymorphism impacts human cingulate-amygdala interactions: A genetic susceptibility mechanism for depression. Nature Neuroscience, 8, 828–834. 10.1038/nn1463. 15880108

[ref45] Poldrack R. A. (2006). Can cognitive processes be inferred from neuroimaging data? Trends in Cognitive Science, 10, 59–63. 10.1016/j.tics.2005.12.004. 16406760

[ref46] Poldrack R. A. (2011). Inferring mental states from neuroimaging data: From reverse inference to large-scale decoding. Neuron, 72, 692–697. 10.1016/j.neuron.2011.11.001.. 22153367 PMC3240863

[ref47] Reuter M. Montag C. (Eds.) 2016). Neuroeconomics. Berlin Heidelberg: Springer.

[ref48] Richiardi J., Altmann A., Milazzo A.-C., Chang C. Chakravarty M. M. (2015). Correlated gene expression supports synchronous activity in brain networks. Science, 348, 1241–1244. 10.1126/science.1255905. 26068849 PMC4829082

[ref49] Sartori S. B., Landgraf R. Singewad N. (2011). The clinical implications of mouse models of enhanced anxiety. Future Neurology, 6, 531–571. 10.2217/fnl.11.34. 21901080 PMC3166843

[ref50] Weimann J. M. Marder E. (1994). Switching neurons are integral members of multiple oscillatory networks. Current Biology, 4, 896–902. 10.1016/S0960-9822(00)00199-8. 7850423

[ref51] van den Berg S. M., de Moor M. H. M., Verweij K. J. H., Krueger R. F., Luciano M., Vasquez A. A., & Boomsma D. I. (2016). Meta-analysis of genome-wide association studies for extraversion: Findings from the genetics of personality consortium. Behavior Genetics, 46, 170–182. https://doi.org/ 10.1007/s10519-015-9735-5. 26362575 10.1007/s10519-015-9735-5 PMC4751159

[ref52] Vul E., Harris C., Winkielman P. Pashler H. (2009). Puzzlingly high correlations in fMRI studies of emotion, personality, and social cognition. Perspective Psychological Science, 4, 274–290. 10.1111/j.1745-6924.2009.01125.x.. 26158964

[ref53] Yarkoni T. (2012). Psychoinformatics: New horizons at the interface of the psychological and computing sciences. Current Directions in Psychological Science, 21, 391–397. 10.1177/0963721412457362.

